# War‐related trauma linked to increased sustained attention to threat in children

**DOI:** 10.1111/cdev.13739

**Published:** 2022-02-11

**Authors:** Julia Michalek, Matteo Lisi, Nicola Binetti, Sumeyye Ozkaya, Kristin Hadfield, Rana Dajani, Isabelle Mareschal

**Affiliations:** ^1^ School of Biological and Behavioural Sciences Queen Mary University of London London UK; ^2^ Department of Psychology Royal Holloway University of London London UK; ^3^ International School for Advanced Studies (SISSA) Trieste Italy; ^4^ School of Psychology Trinity Centre for Global Health Trinity College Dublin Dublin Ireland; ^5^ Department of Biology and Biotechnology Faculty of Science Hashemite University Zarqa Jordan

## Abstract

Experiences of war and displacement can have profound effects on children's affective development and mental health, although the mechanism(s) underlying these effects remain unknown. This study investigated the link between early adversity and attention to affective stimuli using a free‐viewing eye‐tracking paradigm with Syrian refugee (*n *= 31, *M*_age_ = 9.55, 12 female) and Jordanian non‐refugee (*n *= 55, *M*_age_ = 9.98, 30 female) children living in Jordan (March 2020). Questionnaires assessed PTSD, anxiety/depression, insecurity, distress, and trauma. Refugee children showed greater initial avoidance of angry and happy faces compared to non‐refugee children, and higher trauma exposure was linked to increased sustained attention to angry stimuli. These findings suggest that war‐related trauma may have differential effects on the early and later stages of affective processing in refugee children.

## Early adversity and emotion processing

Early adversity, such as childhood maltreatment, is linked to abnormalities in affective processing including altered emotion regulation and recognition (Berzenski, 2018; Pollak, 2012). Children who experienced early adversity often display atypical attention allocation to negative emotional stimuli. For instance, young children who had experienced family violence responded more quickly to angry facial expressions in an emotional dot‐probe task (Briggs‐Gowan et al., 2015), and abused children made fewer errors when responding to anger in a Go/NoGo task (Pollak et al., 2001). Maltreated children also showed heightened attention to angry faces compared to controls, even when instructed to ignore them (Shackman & Pollak, 2007), and physical abuse has been linked to difficulties in disengaging from threatening facial expressions (Pollak & Tolley‐Schell, 2003).

Pollak (2012) proposed that this increased attention to anger may reflect a useful short‐term adaptation in response to adverse and unsafe environments. For example, abusive mothers tend to display atypical facial expressions of anger, which may result in children's heightened responsivity to negative emotions (Shackman et al., 2010). However, this constant vigilance to threat may impair social functioning in the long run and become a potential cause for maladaptive and aggressive behaviors, as well as subsequent psychopathology in childhood and adolescence (Harms et al., 2019). Although the majority of studies report attention *toward* negative stimuli, Pine et al. (2005) report that children who experienced severe domestic abuse displayed attentional *avoidance* of threatening faces. This bias was only observed in physically maltreated but not neglected children, suggesting varying patterns of affective development for different types of childhood adversity. This is consistent with the dimensional model of adversity and psychopathology (DMAP) which proposes that different neurodevelopmental mechanisms are activated by deprivation and by abuse, which could lead to different mental health and behavioral outcomes (McLaughlin et al., 2014). It is also possible that different types of childhood adversity affect different *stages* of information processing (i.e., the initial detection of threat versus the subsequent attention/avoidance of it).

## Psychopathology and atypical emotion processing

Psychopathology is also often associated with atypical emotion processing. For example, anxiety disorders and PTSD are often characterized by increased attention to threat stimuli such as angry faces (Dalgleish et al., 2001; Roy et al., 2008), with studies reporting both hypervigilance and sustained attention (Armstrong & Olatunji, 2012; Dudeney et al., 2015). In line with both the *vigilance* and *maintenance* hypotheses of anxiety (Weierich et al., 2008), these types of attentional biases might increase state and trait anxiety, and contribute to the maintenance of the disorder (Cisler & Koster, 2010).

Children at risk for—or diagnosed with—depression display increased attention toward sad and fearful stimuli (Romens & Pollak, 2012; Tsypes et al., 2017), and neuroimaging studies reveal atypicalities in brain activity during the presentation of sad stimuli in preschool children at risk for the disorder (Barch et al., 2012). The increased maintenance of attention on dysphoric stimuli is consistent with the cognitive model of depression (Beck, 1976) and indicates links to negative affect and maladaptive cognitive patterns present in the disorder (Peckham et al., 2010). However, differences in attentional responses associated with depression in childhood also vary across different studies (Gibb et al., 2016) and these discrepancies suggest that attention patterns may vary depending on the processing stage, the type of stimuli and task, as well as the youth's developmental stage, and their psychopathology.

## Refugee children: linking adversity, mental health, and affective processing

Many child refugees experience a variety of extremely traumatic events (Khamis, 2019), and high rates of war trauma exposure and displacement‐related experiences are a major risk factor for child emotional and behavioral problems (Bryant et al., 2018). Indeed, high rates of posttraumatic stress disorder (PTSD), anxiety, depression, and high psychopathological comorbidity, as well as poorer physical health are regularly reported by refugee children and adolescents (Yayan et al., 2020). Refugee children's adverse experiences may also affect their overall well‐being and psychosocial functioning, as reported using human insecurity and distress scales (e.g., Panter‐Brick, Dajani, et al., 2018; Ziadni et al., 2011).

Although poorer mental health is well documented in refugee children, much less is known about co‐occurring cognitive and affective impairments, which might significantly influence or moderate behavioral and well‐being outcomes in these children. A recent study of Syrian refugee children and adolescents showed a significant link between greater difficulties in emotion regulation and higher trauma exposure and PTSD symptoms (Khamis, 2019). Similarly, a study on Korean refugee adolescents showed that emotion regulation may act as a mediator between early life trauma and mental health (Lee et al., 2020), which emphasizes the impact of war on affective processing. Furthermore, Afghan refugee adolescents with a history of trauma and high levels of PTSD showed impairments in affective working memory capacity (Mirabolfathi et al., 2020), indicating that cognitive processing as well as emotion regulation may be disturbed by early adversity. Taken together, these findings suggest that affective processing in general may be impaired in refugee children, which might in turn play an important role in maintaining the association between trauma exposure and psychopathology.

## This study

Attention biases in children with a history of early stress and mental health problems have been studied extensively, however, this aspect of affective processing has not been explored in displaced refugee children, a group who are at a substantially increased risk for psychopathology. Similarly, although war trauma and displacement negatively influence mental health and well‐being (Yayan et al., 2020), we do not know which cognitive mechanisms link war trauma exposure to this increased vulnerability. Therefore, our aim was to explore the association between war‐related adversity, attentional biases to affective stimuli, and mental health outcomes in 7‐ to 11‐year‐old refugee and non‐refugee children living in Amman, Jordan. Based on previous research, we hypothesized that (1) refugee children will have higher scores on measures of trauma exposure (caregiver‐reported), posttraumatic stress symptoms (PTSS), anxiety/depressive symptoms, insecurity, and distress (all child‐reported) than non‐refugee children; (2) refugee children will spend significantly more time attending to angry and sad facial expressions than non‐refugee children; and 3) the proportion of time spent attending to negative emotional faces will be positively associated with trauma exposure, anxiety/depression, PTSS, insecurity, and distress in all children.

## METHODS

### Participants

Participants (*N *= 86) were Syrian refugee (*n *= 31; *M*_age_ = 9.55, *SD* = 1.84; 12 female) and Jordanian non‐refugee children (*n *= 55; *M*_age_ = 9.98, *SD* = 1.78; 30 female) aged between 7 and 11 years old, living in Amman, Jordan. Children were recruited through a non‐profit organization (We Love Reading, Taghyeer), collaborating with the Zaha Culture Centre in the Al Zohour neighborhood of Amman. Testing took place at the Zaha Culture Centre, which advertised the study to local parents of refugee and non‐refugee children. Caregivers of children taking part in the study were given the trauma events checklist while their children completed the behavioral tasks and the survey measures. Data collection took place in March 2020.

### Demographics, trauma, and psychopathology

Trauma, mental health, and demographics were measured using questionnaires which were originally developed in Arabic or adapted to Arabic. Questionnaires were administered by Arabic‐speaking fieldworkers using the Qualtrics Offline Application on a portable laptop (Qualtrics). Demographic information collected included age, gender, and dependency ratio which was calculated as *number of dependents* / *number of non*‐*dependents* for each household (i.e., number of individuals who do not provide income divided by number of individuals who provide income), and was used as the poverty measure. A higher dependency ratio indicates higher poverty.

War trauma exposure was measured using the 21‐item Traumatic Events Checklist (TEC; Panter‐Brick et al., 2009). Parents answered yes or no about their child's exposure to traumatic events, such as having lived in a refugee camp, having witnessed torture, or having their home forcibly searched by the police or an armed militia. This questionnaire was administered to caregivers about the child's exposure, due to the challenging nature of the questions. Children with TEC scores of 1 and above (i.e., they had experienced at least one traumatic event as reported by the caregiver) were asked if they remembered any traumatic experiences. Those children who were able to recall any traumatic experiences (refugees *n* = 11, non‐refugees *n *= 18) were assessed for PTSS using the Child Revised Impact of Events Scale (CRIES 8; Perrin et al., 2005), administered to the participants. No single traumatic event was specified to the children. Children with TEC scores of 0 (their parent or caregiver indicated that the child had experienced no traumatic events) and those who did not recall any traumatic events were excluded from the measure. CRIES measures symptoms of PTSD with questions relating to a traumatic event, for example, “Do you think about it even if you don't mean to?” and “Do you try to remove it from your memory?”. CRIES is scored on a scale ranging from 0 (*not at all*) to 5 (*often*), with higher scores indicating greater symptom severity (8 items, Cronbach's *α* = .87). The total CRIES score can range from 0 to 40.

Child self‐reports of depression and anxiety symptoms were measured using the Arab Youth Mental Health scale (AYMH; Mahfoud et al., 2011). This measure includes 21 3‐point Likert scale questions (*rarely*, *sometimes*, *always*) and results in a total score for anxiety/depression (Cronbach's *α* = .74), with a higher score indicating more symptoms. Items relate to the child's emotional well‐being in the past week and include statements such as “During the last week I was angry” and “During the last week I was sad.”

The Human Insecurity and Distress Scale (HIDS; Ziadni et al., 2011), which has been previously used with similarly aged children (Michalek et al., 2021), was used to assess two constructs: insecurity (10 items, Cronbach's *α* = .78) and distress (12 items, Cronbach's *α* = .80). HIDS uses a 4‐point Likert scale ranging from 1 (*never*) to 4 (*always*), where higher scores indicate higher rates of insecurity or distress. Human insecurity is akin to fear and includes items such as “To what extent do you worry/fear for your and your family's future?”. Distress refers to unpleasant emotions in response to previous adversity and current difficulties and is measured with items such as “To what extent do you feel frustrated?”. The two subscales were calculated separately, resulting in one score for insecurity and one for distress for each child.

### Attention bias

#### Stimuli & procedure

We used adult and child faces as stimuli but were unable to find a database with images of Arabic children's faces, consequently we used Caucasian faces for both the adult and child stimuli. The stimuli consisted of 16 photographs of four actors: two of the actors were adults (1 female) taken from the Radboud Faces Database (Langner et al., 2010), and two were children (1 female) taken from the Child Affective Facial Expressions (CAFE) database (LoBue & Thrasher, 2015). Each actor expressed four emotions: anger, happiness, sadness, and neutral. We validated the stimuli in an independent convenience sample of Arabic children living in Palestine (*n* = 21, aged 8–10 years old) via an online study using Qualtrics, with good recognition accuracy for all emotional expressions (angry = 90%, happy = 100%, neutral = 73%, sad = 82%), and no significant differences in emotion recognition accuracy between child and adult actors’ expressions (all *p *> .05). The stimuli were in color and displayed on a white background using Matlab (Mathworks, version 2018b) and Psychtoolbox‐3 (Brainard, 1997) routines on two Dell laptop computers with a screen resolution 1366 × 678 (59 participants completed the tasks on a 31 cm screen laptop, and 27 participants completed the task on a 29.5 cm screen laptop). Eye movements were recorded using a Tobii Eye Tracker 4C mounted onto the screen with a sampling rate of 90 hz and children sat approximately 57 cm from the screen without the use of a chin rest.

**FIGURE 1:**
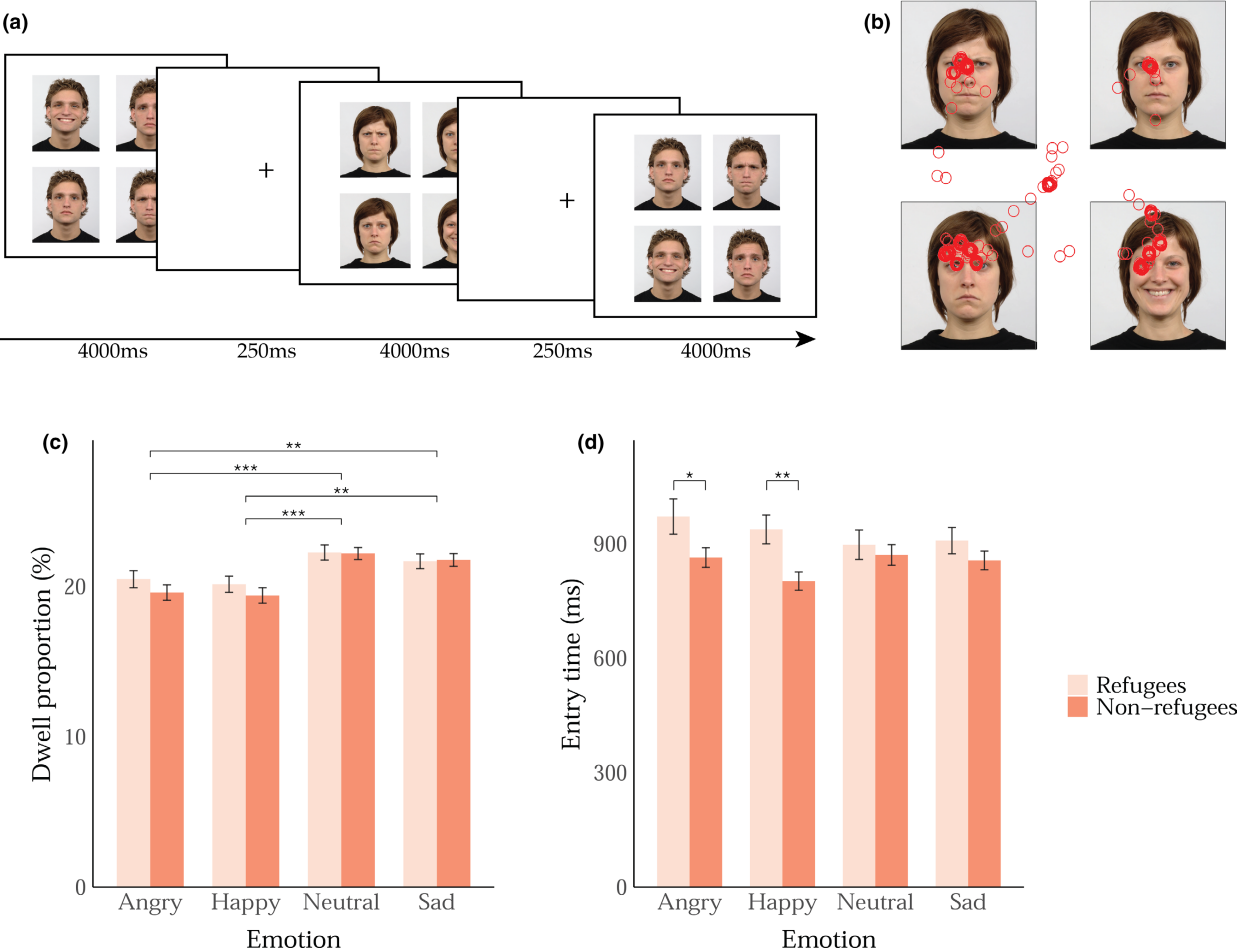
Experimental procedure and data. (a) Three consecutive trials in the adult faces block. (b) Example of gaze position measured during one trial, superimposed on the stimulus for illustrative purposes only. Bar plots of (c) average proportion of dwell and (d) entry time for each emotional expression separated by group. Error bars represent standard error of the mean. *** *p* < .001, ** *p* < .01, * *p *< .05

The eye‐tracking task was a free‐viewing procedure (no response is required from the child) adapted from Bodenschatz et al. (2019). It took approximately 10 min to complete, including instructions and gaze calibration. On each trial of the task, the child saw four same identity faces of an actor displaying the four different emotions. Each image of the actor subtended 5.45 × 8.18 degrees and was displayed in a square 2 × 2 grid in the center of the screen. Trials were randomized in such a way that each emotional expression for each actor appeared in every corner of the grid at least once. The grid location of emotional expressions was pseudo‐randomized, and trials were fully randomized between participants. Each trial began with a fixation cross in the center of the screen for 250 ms, immediately followed by the presentation of the four faces grid displayed for 4000 ms (Figure 1a). The adult and child stimuli were run in separate blocks of 32 trials each and were counterbalanced across children.

Prior to each block, the child's gaze was calibrated using a 9‐point display of small images of dinosaurs to ensure accuracy of the eye gaze recordings. If the calibration was deemed too noisy (by visual inspection), it was repeated until successful. After calibration, the children were instructed to simply look at the images on the screen however they wanted (“free viewing,” no responses required) and to stay as still as possible.

#### Eye gaze processing

Regions of interest (ROIs) were defined using Matlab by drawing four squares around each of the four photographs (face + background), separately for the two testing laptops. Data from the two laptops were combined and we pooled data from ROIs for each emotion across all trials, separately for the adult and child actors. We measured two eye gaze parameters: (1) *dwell*: the proportion of all gaze position samples located within each emotion ROI averaged across the trials, and (2) *entry time*: the average time taken from the start of each trial to the onset of the first 100 ms fixation in each emotion ROI. *Dwell* provides information about sustained attention (late attentional stage) and *entry time* indicates initial attention allocation (early attentional stage). Sample raw gaze data from one child's trial, superimposed on the stimuli from that trial are presented in Figure 1b.

There is usually some data loss with eye‐tracking studies, because of environmental conditions and/or the participant briefly looks off‐screen. In our study, trials with over 50% of missing data (i.e., 11 ms samples falling outside of the computer screen or not recorded by the eye tracker, which comprised 1.6 trials on average across the study) and children with over 50% of missing trials (refugees *n *= 4, non‐refugees *n* = 1) were excluded from the analysis. With presentation time of 4 s the inclusion of a small number of trials with 50% missing data provides 2 s of gaze recordings, which is sufficient for capturing eye movements across all four regions of interest. The number of missing data (invalid samples) of each participant did not vary as a function of group (refugee vs. non‐refugee) or any other variables of interest (all *p *> .05) (Table S7). As there were no significant differences in children's data using either the child or adult actors (entry time and dwell *p *> .05), all subsequent analyses were conducted on the adult and child conditions combined.

### Statistical analysis

To test whether eye gaze patterns differed between refugee and non‐refugee children, we conducted a two‐way mixed‐model ANOVA, with the group as a between‐subjects factor (2 levels: refugees, non‐refugees) and emotion condition as a repeated within‐subjects measure (4 levels: happy, angry, neutral, sad). ANOVAs were performed separately on dwell and entry time and post hoc tests were corrected for multiple testing with the Benjamini–Hochberg false discovery rate (FDR) procedure (Benjamini & Hochberg, 1995).

To compare scores on trauma, PTSD symptoms, internalizing problems, insecurity, and distress between refugees and non‐refugees, the groups were compared using a Mann–Whitney *U* test, as the questionnaire data were not normally distributed and used ordinal scales.

Linear regression analyses were conducted to explore the association between attention patterns (dwell and entry time) and scores on trauma. Separate multiple linear regression models were then fitted with each mental health outcome as a predictor, using the trauma score as a covariate, and attention measures for each emotion as the outcome. Trauma score was included as a covariate in analyses investigating the association between attention and mental health measures given the potential impact of traumatic experiences on children's mental health (e.g., Arakelyan & Ager, 2021). To account for multiple testing, all regression results were corrected using the FDR procedure (Benjamini & Hochberg, 1995). In addition, to establish the likelihood of the alternative hypothesis we used Bayes Factor analyses for each test with JASP (version 0.14.1; JASP Team, 2020) (see Tables S4–S6).

## RESULTS

### Trauma, PTSD, anxiety/depression, insecurity, and distress

Trauma, mental health, and demographic results are presented in Table 1. The two groups did not differ in age or poverty levels (*p *> .05). As expected, we found that refugee children had experienced significantly more trauma compared to non‐refugee children, *n *= 74, *U* = 1119.00, *p *< .001. Caregiver‐reported trauma scores were not significantly correlated with scores on PTSS, anxiety/depression, insecurity, or distress.

**TABLE 1:** Summary of outcomes for refugee and non‐refugee children and results of Mann–Whitney *U* Test of differences between the groups

Characteristic	Refugees				Non‐refugees					
	*n*	*M*	*SD*	*n*		*M*	*SD*	*U*		*p*
Age	31	9.55	1.84	55		9.98	1.78	736.50		.29
Dependency ratio	26	0.23	0.16	52		0.20	0.10	693.00		.86
TEC	26	6.38	3.10	48		1.58	2.03	1119.00		<.001
CRIES	11	6.91	8.20	18		5.78	8.18	105.00		.39
AYMH	25	26.33	3.12	44		24.95	3.81	685.50		.02
HIS	21	20.00	6.21	46		19.65	5.48	491.50		.46
HDS	24	17.63	5.22	51		16.69	4.14	657.00		.31

Abbreviations: AYMH, Arab Youth Mental Health scale (anxiety/depression measure); CRIES, Child Revised Impact of Events Scale (PTSD symptoms measure); HDS, Human Distress Scale; HIS, Human Insecurity Scale; *M*, mean; *SD*, standard deviation; TEC, Traumatic Events Checklist (parent reports).

Among the children who could subjectively recall at least one traumatic event (refugees *n* = 11, non‐refugees *n* = 18), there was no significant difference between refugee and non‐refugee children in PTSS scores, *U *= 105.00, *p *= .39, and most children who remembered exposure to trauma did not report any PTSD symptoms (59%, *n* = 17).

Refugee children reported more anxiety and depression symptoms compared to non‐refugee children, *n *= 68, *U *= 685.50, *p *= .021, which remained significant after correcting for multiple comparisons (*p *< .04). There were no significant differences between refugees and non‐refugees in terms of insecurity (*n *= 67, *U *= 491.50, *p *= .46) or distress (*n* = 75, *U *= 657.00, *p *= .31).

### Dwell

Mauchly's test indicated that the assumption of sphericity had been violated, *χ* ^2^(5) = 20.71, *p* < .001, Greenhouse Geisser *ε *= .85, and therefore results are reported using Huynh–Feldt correction (*ε* = .89). One outlier >3 SD from the mean was excluded from analysis. The 2 × 4 (group × emotion) mixed model ANOVA on dwell showed a significant main effect of emotion, *F*(2.67, 205.30) = 11.28, *p *< .001, *η*_p_^2^ = .13, suggesting that the proportion of dwell varied for different emotions. There was no main effect of group, *F*(1, 77) = .71, *p *= .40, *η*_p_^2^ = .01, or group × emotion interaction, *F*(2.67, 205.30) = .41, *p *= .72, *η*_p_^2^ = .01, indicating that the proportion of dwell on the different emotions did not differ between refugee and non‐refugee children. Post hoc tests revealed significant differences in dwell between angry—neutral (*p *< .001), angry—sad (*p* = .004), happy—neutral (*p* < .001), and happy—sad (*p *= .001), revealing that both refugee and non‐refugee children spent less time looking at angry and happy faces as compared to sad and neutral faces (Figure 1c & Table S1). These results remained significant after FDR corrections.

**FIGURE 2:**
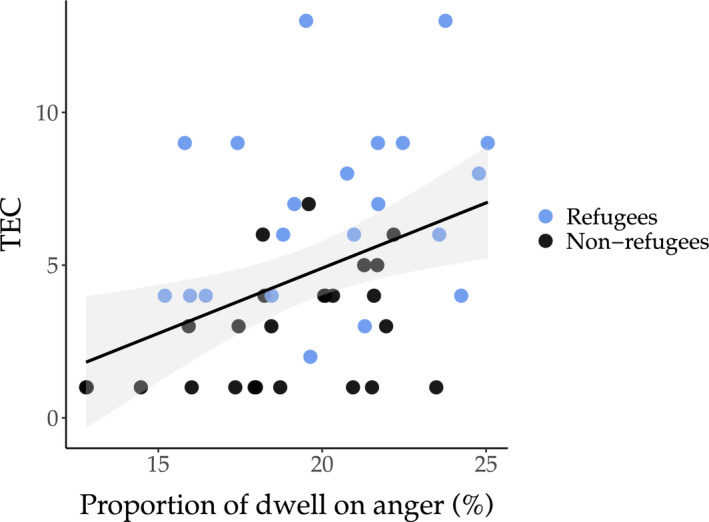
Trauma and sustained attention to threat. Association between traumatic events experienced (TEC) and proportion of dwell on anger

Linear regression analysis (Table S2) showed that proportion of dwell on anger was significantly predicted by trauma scores, *F*(1, 44) = 8.66, *p *= .005, *b *= .39, *R* ^2^ = .164, indicating that the children with higher trauma scores spent more time attending to angry faces (Figure 2). This result remained significant after FDR multiple testing corrections. Scores for symptoms of PTSD, internalizing problems, insecurity, and distress (with trauma score as a covariate) did not significantly predict dwell on any of the emotions (Table S2).

### Entry time

Two outliers >3 SD from the mean were excluded from the analysis. There was no significant effect of emotion on entry time, *F*(3, 228) = 1.85, *p *= .14, *η*_p_^2^ = .02. However, there was a significant effect of group, *F*(1, 76) = 4.09, *p *= .047, *η*_p_^2^ = .05, revealing that refugee children took longer to attend to the stimuli than non‐refugee children. There was also a significant group x emotion interaction, *F*(3, 228) = 3.06, *p* = .029, *η*_p_^2^ = .04, indicating that entry times from refugee and non‐refugee participants were different for different emotions. Post hoc tests revealed differences between refugees and non‐refugees in entry time for anger (*p *= .033, *η*_p_^2^ = .06) and happiness (*p *= .003, *η*_p_^2^ = .12); refugee children were slower to look at anger and happiness than non‐refugee children (Figure 1d & Table S3). These results remained significant after FDR corrections (*p *< .05 and *p* < .025, respectively).

Linear regression analyses showed that trauma, internalizing problems, insecurity, and distress did not significantly predict entry time on any emotions (Table S2).

## DISCUSSION

The aims of this study were threefold. First, to investigate if early adversity negatively impacts mental health in displaced refugee children in Jordan; second, to examine if refugee children attend to negative faces more than non‐refugee children; and third, to measure whether increased attention to negative emotional faces was associated with poorer mental health and higher trauma exposure across all children.

As predicted, we found that refugee children experienced more traumatic events related to war and displacement than non‐refugee children. Refugee children also reported more internalizing problems, related to anxiety and depression, than non‐refugee children. Surprisingly, they had lower scores for PTSD symptoms severity, as well as for feelings of insecurity and distress than previously reported using the same measures in adolescents (e.g., Panter‐Brick, Dajani, et al., 2018; Panter‐Brick, Hadfield, et al., 2018) and these scores did not differ between our groups. There are a few reasons why the refugee children may have had lower symptom severity than previously reported. Firstly, Khamis (2019) reports that time spent living in a host country significantly reduces PTSD symptoms in Syrian refugee children and adolescents. Furthermore, while mental health outcomes were reported by the children, trauma was reported by the caregiver, and many children did not recall the caregiver‐reported traumatic events. Considering that, for instance, pediatric PTSD has been previously linked to trauma memory (e.g., McGuire et al., 2021), the lack of conscious trauma recall in most of the children might explain the lack of group differences in PTSD symptoms, as well as the lack of associations between parent‐reported trauma scores and child‐reported psychopathology. We also did not investigate the severity of trauma reported, which might influence both affective processing and trauma recall. Finally, previous studies mainly investigated mental health in refugee children living in refugee camps (e.g., Sirin & Rogers‐Sirin, 2015), whereas we have focused on those living outside of refugee camps. We have chosen to focus on youth outside refugee camps because 83% of refugees in Jordan live in urban areas (UNHCR, 2021), highlighting the importance of the current environment in children's development. Taken together, our results suggest that time passed since the experience of war‐related events and current living conditions may mitigate the effects of early war‐related adversity on children's development. This seems consistent with recent findings in non‐refugee groups that conscious, subjective recall of early life trauma is a more important factor in later mental health problems than objective records of exposure (Danese & Widom, 2020; Rivenbark et al., 2020).

Contrary to our second hypothesis, we found no differences between refugee and non‐refugee children in their sustained attention (dwell time) to the emotional faces. However, both groups of children spent more time attending to neutral and sad faces compared to angry and happy faces. In this, our results differ from previous studies using different paradigms, which report that children with a history of adversity display an attention bias *toward* threatening stimuli (Berzenski, 2018; Briggs‐Gowan et al., 2015). The similarities in dwell on the emotional faces in refugee and non‐refugee children may reflect the lack of group differences we found in mental health measures and poverty. This suggests that other factors might affect emotion processing more than war‐related trauma, emphasizing the differential influence of adversity types on outcomes such as cognitive processing and mental health (McLaughlin et al., 2014). Indeed, children exposed to different types of abuse (physical, sexual, and neglect) can display different types of impairments in emotion processing, reinforcing the idea that adversity does not have a general effect on affective processes (Pfaltz et al., 2019).

Although there were no group differences for overall attention to threat stimuli as measured through dwell, we found that refugee children displayed increased initial avoidance of angry and happy faces, compared to non‐refugee children. Threat avoidance in children after early adversity has been reported previously (Pine et al., 2005), although most findings suggest hypervigilance to threat (Briggs‐Gowan et al., 2015). It is possible that the initial avoidance to angry and happy stimuli in our refugee group reflects differences in the saliency or intensity of these expressions as compared to neutral and sad faces (e.g., a general initial aversion to *intense* facial expressions). Another possibility might be that children were able to rapidly deploy their covert attention to the emotional stimuli when they initially appeared on the screen, and then avoided the stimulus during overt attention allocation. Studies with adult participants suggest that angry faces can be accurately detected in parafoveal vision within the first 150 ms of presentation in adults (Calvo et al., 2006), although it is not clear if the same patterns can be observed in children.

As predicted in our third hypothesis, children who experienced more trauma spent more time attending to angry faces (longer dwell), irrespective of refugee status. Longer sustained dwell on the angry faces in children with higher trauma might suggest disengagement difficulties. Our findings are consistent with previous literature which highlights the effects of early adversity on increased attention to threatening stimuli (Briggs‐Gowan et al., 2015; McCoy et al., 2016), and could represent a detrimental cognitive strategy leading to future emotional problems. Overall, our findings highlight differences in early and later processing stages, suggesting a differential impact of war trauma on initial orienting versus sustained attention to emotional stimuli. Further studies should explore attention biases in young refugee children as vulnerability factors for subsequent psychopathology.

Several limitations should be considered when interpreting our findings. First, low scores on mental health scales differentiate the current cohort from some other studies of refugee children and adolescents (Ozer et al., 2016). Although these differences might be explained by the living conditions of children in our study (resettled rather than living in a camp), this could also reflect the resilience or protective factors, which we did not measure (Zwi et al., 2018). Individual, relational, and contextual resilience has been linked to better mental health outcomes in Syrian refugee youth (Panter‐Brick, Hadfield, et al., 2018), and could also play a role in children's affective processing. Indeed, caregiver's trauma, psychopathology, and parenting practices affect children's mental health outcomes, cognitive development, and emotion regulation, both postadoption as well as in refugee settings (Bryant et al., 2020; Eruyar et al., 2018; Koss et al., 2020).

Second, although our findings suggest that children with higher trauma exposure may have trouble disengaging from threat, we were unable to investigate the specific mechanisms driving this process. Attentional control theory proposes that difficulties in disengaging from threat are regulated by impairments in inhibitory mechanisms, suggesting general disruptions to cognitive control (Eysenck et al., 2007). In fact, impairments in effortful control have been previously linked to attention bias to threat in healthy, non‐abused children (Lonigan & Vasey, 2009), as well as to early deprivation experiences (Machlin et al., 2019). Disengagement difficulties might also be moderated by emotion dysregulation (e.g., inability to regulate emotional state by employing threat avoidance) and disturbances in emotion regulation strategies have been previously linked to both early adversity and psychopathology (Heleniak et al., 2016). Recent studies find similar emotion regulation atypicalities in war‐affected refugee children and adolescents (Khamis, 2019; Lee et al., 2020). Future studies should include measures of emotion regulation strategies, as well as measures of general cognitive control, to evaluate the influence of these closely related mechanisms on attention processing.

Third, anxiety disorder symptoms are usually associated with biases for anger or threatening stimuli, whereas depressive disorders have been linked to biases to sad stimuli in children living in high‐income contexts (e.g., Roy et al., 2008; Tsypes et al., 2017). However, the mental health measure used to assess anxiety and depression (AYMH) does not separate the symptoms of these two disorders, and instead provides a combined measure of internalizing problems. Therefore, the current study did not have the capacity to look more finely at the association between individual negative emotional stimuli (e.g., anger and sadness) and separate psychological states. Further research could separate these constructs to identify a more specific set of symptoms and behavioral traits which might be related to affective processing.

Furthermore, the lack of group differences in PTSD symptoms might reflect the fact that the CRIES measure in the current study pertains to *any* traumatic event. Due to the distressing nature of the questionnaire and the young age of the children, we gathered information on their PTSD symptoms only about a traumatic event they remembered. It is possible that refugee children reported their PTSS about a relatively un‐traumatic experience, while experiences of PTSS relating to other traumatic events were not captured. This would artificially decrease their PTSD symptoms making them more similar in their reports to the non‐refugee children. Given our small sample of children with PTSD, we are limited in interpreting the lack of association between trauma and PTSD in the current study. Moreover, due to small sample size overall we were unable to further explore the effects that different types of trauma may have on attention to emotional stimuli and mental health outcomes.

Finally, due to the lack of availability of Arabic faces databases at the time of the study, we used Caucasian faces which might be less common to our sample of children. However, we were interested in comparing performance between groups of Arabic children, so this potential effect of test face ethnicity should be minimal.

In conclusion, we found that refugee children displayed greater initial avoidance toward angry and happy stimuli than non‐refugees and that those children who experienced more trauma dwelled on anger longer. Our findings highlight the link between war‐related trauma and emotion processing in refugee children and emphasize the difference between early and later attention stages in affective processing after adversity.

## Ethics

The project was granted ethical approval from the Queen Mary University of London research ethics board in 2018 (QMERC2018/54). Parents or guardians gave their informed consent prior to children taking part. Before and during the testing, children were asked if they were happy to proceed and informed that they could stop at any time. Each child was given a sleeve of stickers, juice, and 4JOD for travel costs for the child's family.

## CONFLICT OF INTEREST

All authors declare no competing interests.

## Supporting information

Table S1‐S7
