# Supplementary material for: War‐related trauma linked to increased sustained attention to threat in children
Source: Child Dev. 2022 Feb 11;93(4):900–9. doi: 10.1111/cdev.13739 (PMC9542223; doi:10.1111/cdev.13739)
Supplement: Supplementary file 1 — Table S1‐S7 [file CDEV-93-900-s001.docx]

**Supplemental material**

**Table 1.**

*Means, standard errors, and confidence intervals for the proportion of dwell duration (%) on anger, happy, neutral, and sad stimuli*

| Emotion | *M* | *SE* | 95% Confidence Intervals | |
| --- | --- | --- | --- | --- |
|  |  |  | Lower Bound | Upper Bound |
| Anger | 20.04 | .418 | 19.20 | 20.87 |
| Happy | 19.82 | .413 | 18.99 | 20.64 |
| Neutral | 22.24 | .341 | 21.56 | 22.91 |
| Sad | 21.69 | .353 | 20.98 | 22.39 |

**Table 2.**

*Linear regression models of dwell and entry time for each emotion.*

| ***Dwell (sustained attention)*** | | | | | | | |
| --- | --- | --- | --- | --- | --- | --- | --- |
|  | *t* | *p* | *b* | *F* | *df* | *p* | *adj.R^2^* |
| Anger |  |  |  |  |  |  |  |
| 1.Overall model |  |  |  | 8.66 | 1,44 | .005 | .15 |
| TEC | 2.94 | .005 | .39 |  |  |  |  |
| 2. Overall model |  |  |  | 0.20 | 2, 7 | .82 | -.22 |
| CRIES | -0.57 | .584 | -.22 |  |  |  |  |
| TEC | -0.25 | .813 | -.10 |  |  |  |  |
| 3. Overall model |  |  |  | 2.73 | 2, 32 | .080 | .09 |
| AYMHS | -0.08 | .933 | -.01 |  |  |  |  |
| TEC | 2.33 | .026 | .33 |  |  |  |  |
| 4. Overall model |  |  |  | 4.70 | 2, 32 | .016 | .18 |
| HIS | 1.49 | .146 | .16 |  |  |  |  |
| TEC | 2.43 | .021 | .26 |  |  |  |  |
| 5. Overall model* |  |  |  | 4.31 | 2, 37 | .021 | .15 |
| HDS | 0.10 | .920 | .01 |  |  |  |  |
| TEC | 2.87 | .007 | .39 |  |  |  |  |
| Happy |  |  |  |  |  |  |  |
| 1.Overall model |  |  |  | 1.89 | 1,45 | .176 | .02 |
| TEC | 1.37 | .176 | .21 |  |  |  |  |
| 2. Overall model |  |  |  | 0.60 | 2, 8 | .571 | -.09 |
| CRIES | -0.22 | .834 | -.08 |  |  |  |  |
| TEC | 1.08 | .310 | .46 |  |  |  |  |
| 3. Overall model |  |  |  | 0.40 | 2, 33 | .671 | -.04 |
| AYMHS | -0.68 | .501 | -.11 |  |  |  |  |
| TEC | 0.64 | .526 | .11 |  |  |  |  |
| 4. Overall model |  |  |  | 0.81 | 2, 32 | .453 | -.01 |
| HIS | 0.44 | .663 | .06 |  |  |  |  |
| TEC | 1.12 | .272 | .20 |  |  |  |  |
| 5. Overall model |  |  |  | 0.79 | 2, 37 | .459 | -.01 |
| HDS | 0.48 | .635 | .05 |  |  |  |  |
| TEC | 1.07 | .294 | .18 |  |  |  |  |
| Neutral |  |  |  |  |  |  |  |
| 1.Overall model |  |  |  | 2.21 | 1,45 | .144 | .03 |
| TEC | 1.49 | .144 | .19 |  |  |  |  |
| 2. Overall model |  |  |  | 0.96 | 2, 8 | .424 | -.01 |
| CRIES | 0.01 | .991 | <.01 |  |  |  |  |
| TEC | -1.38 | .204 | -.23 |  |  |  |  |
| 3. Overall model |  |  |  | 1.62 | 2, 33 | .214 | .03 |
| AYMHS | -0.99 | .330 | -.13 |  |  |  |  |
| TEC | 1.58 | .124 | .24 |  |  |  |  |
| 4. Overall model |  |  |  | 1.14 | 2, 32 | .332 | .01 |
| HIS | 0.30 | .763 | .04 |  |  |  |  |
| TEC | 1.42 | .166 | .22 |  |  |  |  |
| 5. Overall model |  |  |  | 1.49 | 2, 37 | .239 | .02 |
| HDS | -0.76 | .451 | -.07 |  |  |  |  |
| TEC | 1.66 | .106 | .23 |  |  |  |  |
| Sad |  |  |  |  |  |  |  |
| 1.Overall model |  |  |  | 0.54 | 1, 45 | .468 | -.01 |
| TEC | 0.73 | .468 | .12 |  |  |  |  |
| 2. Overall model |  |  |  | 0.20 | 2, 8 | .820 | -.19 |
| CRIES | 0.12 | .911 | .02 |  |  |  |  |
| TEC | 0.62 | .551 | .14 |  |  |  |  |
| 3. Overall model |  |  |  | 0.24 | 2, 33 | .790 | -.05 |
| AYMHS | -0.43 | .671 | -.06 |  |  |  |  |
| TEC | 0.57 | .570 | .09 |  |  |  |  |
| 4. Overall model |  |  |  | 0.39 | 2, 32 | .680 | -.04 |
| HIS | 0.57 | .576 | .07 |  |  |  |  |
| TEC | 0.59 | .560 | .10 |  |  |  |  |
| 5. Overall model |  |  |  | 0.53 | 2, 37 | .590 | -.03 |
| HDS | -0.65 | .519 | -.07 |  |  |  |  |
| TEC | 0.90 | .373 | .13 |  |  |  |  |
| ***Entry time (initial orienting)*** | | | | | | | |
|  | *t* | *p* | *b* | *F* | *df* | *p* | *adj.R^2^* |
| Anger |  |  |  |  |  |  |  |
| 1.Overall model |  |  |  | 0.42 | 1, 43 | .519 | -.01 |
| TEC | 0.65 | .649 | .61 |  |  |  |  |
| 2. Overall model |  |  |  | 2.78 | 2, 8 | .121 | .26 |
| CRIES | -0.84 | .423 | -1.42 |  |  |  |  |
| TEC | 2.24 | .056 | 4.09 |  |  |  |  |
| 3. Overall model |  |  |  | 0.61 | 2, 31 | .549 | -.02 |
| AYMHS | 0.85 | .400 | .84 |  |  |  |  |
| TEC | 0.67 | .507 | .72 |  |  |  |  |
| 4. Overall model |  |  |  | 0.38 | 2, 30 | .690 | -.04 |
| HIS | 0.47 | .643 | .37 |  |  |  |  |
| TEC | 0.66 | .512 | .71 |  |  |  |  |
| 5. Overall model |  |  |  | 0.41 | 2, 35 | .668 | -.03 |
| HDS | 0.76 | .452 | .52 |  |  |  |  |
| TEC | 0.35 | .730 | .35 |  |  |  |  |
| Happy |  |  |  |  |  |  |  |
| 1.Overall model |  |  |  | 3.45 | 1, 43 | .070 | .05 |
| TEC | 1.86 | .070 | 1.63 |  |  |  |  |
| 2. Overall model |  |  |  | 2.22 | 2, 8 | .171 | .20 |
| CRIES | -0.22 | .484 | -1.00 |  |  |  |  |
| TEC | 2.01 | .080 | 2.96 |  |  |  |  |
| 3. Overall model |  |  |  | 1.05 | 2, 31 | .361 | <.01 |
| AYMHS | 0.11 | .916 | .09 |  |  |  |  |
| TEC | 1.44 | .159 | 1.40 |  |  |  |  |
| 4. Overall model |  |  |  | 1.07 | 2, 30 | .356 | <.01 |
| HIS | -0.45 | .658 | -.31 |  |  |  |  |
| TEC | 1.44 | .161 | 1.36 |  |  |  |  |
| 5. Overall model |  |  |  | 1.08 | 2, 35 | .351 | <.01 |
| HDS | 0.47 | .640 | .29 |  |  |  |  |
| TEC | 1.29 | .206 | 1.15 |  |  |  |  |
| Neutral |  |  |  |  |  |  |  |
| 1.Overall model |  |  |  | <0.01 | 1, 43 | .962 | -.02 |
| TEC | 0.05 | .962 | .04 |  |  |  |  |
| 2. Overall model |  |  |  | 2.27 | 2, 8 | .166 | .20 |
| CRIES | -0.13 | .903 | -.18 |  |  |  |  |
| TEC | 2.13 | .066 | 3.34 |  |  |  |  |
| 3. Overall model |  |  |  | 1.05 | 2, 31 | .363 | <.01 |
| AYMHS | 1.45 | .159 | 1.17 |  |  |  |  |
| TEC | 0.02 | .984 | .02 |  |  |  |  |
| 4. Overall model |  |  |  | 0.03 | 2, 30 | .970 | -.07 |
| HIS | -0.03 | .975 | -.02 |  |  |  |  |
| TEC | -0.24 | .814 | -.22 |  |  |  |  |
| 5. Overall model |  |  |  | 0.38 | 2, 35 | .383 | <-.01 |
| HDS | 1.32 | .195 | .78 |  |  |  |  |
| TEC | -0.70 | .490 | -.60 |  |  |  |  |
| Sad |  |  |  |  |  |  |  |
| 1.Overall model |  |  |  | 0.09 | 1, 43 | .761 | -.02 |
| TEC | 0.31 | .761 | .24 |  |  |  |  |
| 2. Overall model |  |  |  | 1.55 | 2, 8 | .270 | .10 |
| CRIES | 0.68 | .518 | 1.03 |  |  |  |  |
| TEC | 1.59 | .150 | 2.62 |  |  |  |  |
| 3. Overall model |  |  |  | 0.19 | 2, 31 | .824 | -.05 |
| AYMHS | -0.54 | .595 | -.44 |  |  |  |  |
| TEC | 0.34 | .738 | .30 |  |  |  |  |
| 4. Overall model |  |  |  | 0.65 | 2, 30 | .529 | -.02 |
| HIS | -0.99 | .332 | -.59 |  |  |  |  |
| TEC | 0.70 | .492 | .57 |  |  |  |  |
| 5. Overall model |  |  |  | 0.08 | 2, 35 | .927 | -.05 |
| HDS | 0.29 | .772 | .16 |  |  |  |  |
| TEC | 0.20 | .373 | .16 |  |  |  |  |

*Note*. TEC = Traumatic Events Checklist (parental reports), CRIES = Child Revised Impact of Events Scale (PTSD symptoms measure), AYMHS = Arab Youth Mental Health Scale (anxiety/depression measure), HIS = Human Insecurity Scale, HDS = Human Distress Scale.

**Table 3.**

*Means, standard errors and univariate analysis of variance results for group differences of entry times (ms) on anger, happy, neutral, and sad faces.*

| Emotion | Refugees | | Non-refugees | | *F*(1,76) | *p* | Partial η^2^ |
| --- | --- | --- | --- | --- | --- | --- | --- |
|  | *M* | *SE* | *M* | *SE* |  |  |  |
| Anger | 88.29 | 3.64 | 78.52 | 2.58 | 4.79 | .033 | .059 |
| Happy | 85.23 | 3.12 | 73.67 | 2.20 | 9.24 | .003 | .108 |
| Neutral | 81.58 | 3.48 | 79.62 | 2.46 | .21 | .648 | .003 |
| Sad | 82.56 | 3.13 | 77.85 | 2.21 | 1.51 | .223 | .025 |

**Table 4.**

*Bayesian Mann – Whitney tests for estimating group differences in demographics, trauma, and mental health*.

| Variable | Group | *Bayes Factor* | *U* | *Mean* | *SE* | 95% Credible Intervals | |
| --- | --- | --- | --- | --- | --- | --- | --- |
|  |  |  |  |  |  | Lower | Upper |
| Age |  | 0.26 | 736.50 |  |  |  |  |
|  | Refugee |  |  | 9.55 | 0.33 | 8.87 | 10.22 |
|  | Non-refugee |  |  | 9.98 | 0.23 | 9.50 | 10.46 |
| Poverty |  | 0.39 | 693.00 |  |  |  |  |
|  | Refugee |  |  | 0.23 | 0.03 | 0.17 | 0.30 |
|  | Non-refugee |  |  | 0.20 | 0.01 | 0.18 | 0.24 |
| TEC |  | 1252.50 | 1119.00 |  |  |  |  |
|  | Refugee |  |  | 6.38 | 0.61 | 5.13 | 7.64 |
|  | Non-refugee |  |  | 1.58 | 0.29 | 0.99 | 2.17 |
| CRIES |  | 0.43 | 105.00 |  |  |  |  |
|  | Refugee |  |  | 6.91 | 2.47 | 1.40 | 12.42 |
|  | Non-refugee |  |  | 5.78 | 1.93 | 1.71 | 9.85 |
| AYMHS |  | 1.30 | 685.50 |  |  |  |  |
|  | Refugee |  |  | 26.33 | 0.64 | 25.02 | 27.65 |
|  | Non-refugee |  |  | 24.95 | 0.57 | 23.80 | 26.11 |
| HIS |  | 0.30 | 491.50 |  |  |  |  |
|  | Refugee |  |  | 20.00 | 1.36 | 17.17 | 22.83 |
|  | Non-refugee |  |  | 19.65 | 0.81 | 18.02 | 21.28 |
| HDS |  | 0.38 | 657.00 |  |  |  |  |
|  | Refugee |  |  | 17.63 | 1.07 | 15.42 | 19.83 |
|  | Non-refugee |  |  | 16.69 | 0.58 | 15.52 | 17.85 |

*Note*. TEC = Traumatic Events Checklist (parental reports), CRIES = Child Revised Impact of Events Scale (PTSD symptoms measure), AYMHS = Arab Youth Mental Health Scale (anxiety/depression measure), HIS = Human Insecurity Scale, HDS = Human Distress Scale.

**Table 5.**

*Bayesian ANOVA models for attention measures differences between the groups and the emotions.*

| ANOVA model | *Bayes Factor* | *Mean* | *SD* | *95% Credible Intervals* | |
| --- | --- | --- | --- | --- | --- |
|  |  |  |  | *Lower* | *Upper* |
| Dwell |  |  |  |  |  |
| Main effect of group | 0.22 |  |  |  |  |
| Refugee |  | 0.18 | 0.23 | -0.28 | 0.62 |
| Non-refugee |  | -0.18 | 0.23 | -0.65 | 0.26 |
| Main effect of emotion | 2.199e+6 |  |  |  |  |
| Angry |  | -0.94 | -.28 | -1.50 | -0.38 |
| Happy |  | -1.10 | 0.28 | -1.66 | -0.54 |
| Neutral |  | 1.27 | 0.28 | 0.81 | 1.82 |
| Sad |  | 0.76 | 0.27 | 0.21 | 1.31 |
| *Post hoc* comparisons |  |  |  |  |  |
| Angry - Happy | 0.13 |  |  |  |  |
| Angry – Neutral | 4873.28 |  |  |  |  |
| Angry – Sad | 124.33 |  |  |  |  |
| Happy – Neutral | 16587.66 |  |  |  |  |
| Happy – Sad | 357.38 |  |  |  |  |
| Neutral - Sad | 0.38 |  |  |  |  |
| Group x emotion interaction | 0.07 |  |  |  |  |
| Entry time |  |  |  |  |  |
| Main effect of group | 1.59 |  |  |  |  |
| Refugee |  | 31.32 | 18.18 | -4.36 | 67.18 |
| Non-refugee |  | -31.32 | 18.18 | -68.93 | 3.80 |
| Main effect of emotion | 0.23 |  |  |  |  |
| Angry |  | 21.11 | 11.20 | -1.12 | 43.41 |
| Happy |  | -18.61 | 11.56 | -41.97 | 3.98 |
| Neutral |  | 2.03 | 11.39 | -20.95 | 23.81 |
| Sad |  | -4.53 | 10.96 | -27.22 | 17.19 |
| Group x emotion interaction | 1.41 |  |  |  |  |
| Angry | 1.86 |  |  |  |  |
| Refugee |  | 11.38 | 10.99 | -10.18 | 33.49 |
| Non-refugee |  | -11.38 | 10.99 | -33.68 | 9.99 |
| Happy | 14.66 |  |  |  |  |
| Refugee |  | 21.00 | 11.24 | -1.19 | 43.67 |
| Non-refugee |  | -21.00 | 11.24 | -43.88 | 0.98 |
| Neutral | 0.28 |  |  |  |  |
| Refugee |  | -22.42 | 11.29 | -45.42 | -0.25 |
| Non-refugee |  | 22.42 | 11.29 | 0.06 | 45.23 |
| Sad | 0.47 |  |  |  |  |
| Refugee |  | -9.96 | 10.77 | -31.93 | 11.20 |
| Non-refugee |  | 9.96 | 10.77 | -11.40 | 31.74 |

*Note. Mean* and *SD* represent the mean and Standard Deviation of the posterior distribution of each parameter.

**Table 6.**

*Bayesian linear regression results for associations between attention measures and questionnaire outcomes.*

|  | TEC | | | | | | | CRIES | | | | | AYMHS | | | | | | | HIS | | | | | HDS | | | | | |
| --- | --- | --- | --- | --- | --- | --- | --- | --- | --- | --- | --- | --- | --- | --- | --- | --- | --- | --- | --- | --- | --- | --- | --- | --- | --- | --- | --- | --- | --- | --- |
|  | *BF* | *M* | *SD* | 95% CL | | *BF* | *M* | | *SD* | 95% CL | | *BF* | | *M* | *SD* | 95% CL | | *BF* | *M* | | *SD* | 95% CL | | *BF* | | *M* | *SD* | 95% CL | |  |
|  |  |  |  | L | U |  |  | |  | L | U |  | |  |  | L | U |  |  | |  | L | U |  | |  |  | L | U |  |
| Dwell |  |  |  |  |  |  |  | |  |  |  |  | |  |  |  |  |  |  | |  |  |  |  | |  |  |  |  |  |
| Anger | 8.20 | .30 | .16 | .00 | .54 | 0.56 | -.01 | | .21 | -.73 | .65 | 0.33 | | -.01 | .07 | -.18 | .18 | 1.04 | .08 | | .10 | -.01 | .34 | 0.35 | | .01 | .05 | -.13 | .13 |  |
| Happy | 0.62 | .07 | .12 | -.07 | .34 | 0.48 | -.02 | | .26 | -.80 | .69 | 0.38 | | -.02 | .08 | -.25 | .08 | 0.38 | .02 | | .06 | -.10 | .21 | 0.38 | | .01 | .05 | -.08 | .18 |  |
| Neutral | 0.71 | .06 | .11 | -.04 | .32 | 0.48 | .01 | | .07 | -.21 | .24 | 0.43 | | -.03 | .08 | -.24 | .09 | 0.36 | .01 | | .06 | -.07 | .17 | 0.34 | | -.01 | .05 | -.14 | .06 |  |
| Sad | 0.36 | .02 | .08 | -.12 | .25 | 0.49 | .04 | | .17 | -.40 | .53 | 0.34 | | -.01 | .06 | -.18 | .09 | 0.39 | .01 | | .06 | -.09 | .19 | 0.34 | | -.01 | .05 | -.16 | .06 |  |
| Entry time |  |  |  |  |  |  |  | |  |  |  |  | |  |  |  |  |  |  | |  |  |  |  | |  |  |  |  |  |
| Anger | 0.35 | .13 | .48 | -.39 | 1.63 | 0.42 | -1.42 | | 2.73 | -9.45 | 4.63 | 0.45 | | .17 | .53 | -.27 | 1.88 | 0.38 | .08 | | .36 | -.48 | 1.38 | 0.42 | | .10 | .34 | -.31 | 1.20 |  |
| Happy | 1.16 | .72 | .89 | -.12 | 2.51 | 0.42 | -.75 | | 2.16 | -7.30 | 3.87 | 0.33 | | .01 | .40 | -.78 | 1.30 | 0.34 | -.05 | | .33 | -.91 | .62 | 0.38 | | .07 | .30 | -.50 | .86 |  |
| Neutral | 0.30 | .01 | .27 | -.78 | .88 | 0.35 | -.30 | | 1.89 | -5.08 | 5.14 | 0.78 | | .33 | .61 | -.04 | 2.18 | 0.33 | -.01 | | .27 | -.68 | .63 | 0.57 | | .19 | .39 | -.12 | 1.38 |  |
| Sad | 0.31 | .05 | .35 | -.78 | .94 | 0.45 | -.32 | | 1.61 | -5.56 | 4.08 | 0.37 | | -.07 | .36 | -1.16 | .60 | 0.46 | .10 | | .39 | -.66 | 1.15 | 0.33 | | .03 | .22 | -.43 | .62 |  |

*Note.* *BF* = Bayes Factor; *M* and *SD* represent the mean and standard deviation of the posterior distribution of each parameter.; 95% CL = 95% Credible Intervals; L = Lower; U = Upper; TEC = Traumatic Events Checklist; CRIES = Child Revised Impact of Events Scale (PTSD symptoms measure), AYMHS = Arab Youth Mental Health Scale (anxiety/depression measure), HIS = Human Insecurity Scale, HDS = Human Distress Scale.

**Table 7.**

*Associations between missing data and variables of interest*

| Variable | *r* | *p* | *n* |
| --- | --- | --- | --- |
| TEC | -.180 | .207 | 51 |
| CRIES | -.354 | .120 | 12 |
| AYMHS | .098 | .179 | 16 |
| HIS | .009 | .944 | 67 |
| HDS | -.036 | .757 | 75 |
| poverty | -.171 | .116 | 86 |
| age | .089 | .437 | 78 |

TEC = Traumatic Events Checklist (parent reports), CRIES = Child Revised Impact of Events Scale (PTSD symptoms measure), AYMHS = Arab Youth Mental Health Scale (anxiety/depression measure), HIS = Human Insecurity Scale, HDS = Human Distress Scale.
